# James W. White, A.M., M.D., D.D.S.

**Published:** 1891-07

**Authors:** 


### Obituary.

### JAMES W. WHITE, A.M., M.D., D.D.S.

Dr. White was born in Bucks County, Pa., in 1826, and was a descendant of Henry White, who settled in Virginia in 1645.

He began his business career May 13, 1841, entering the laboratory of his uncle, Samuel W. Stockton. The latter was the first, as far as known, to enter into the manufacture of artificial teeth as a business. His teeth had a wide reputation, and the manufacture rapidly developed into one of great importance. In this establishment we believe the originals of the S. S. White Manufacturing Company, Jones, White, and McCurdy, were also trained.

The origin of the present great house was at Seventh and Race Streets, Philadelphia, in a comparatively humble building. Here in the attic James W. White began the manufacture of teeth. This work was all done by himself and necessarily crude. He states that the fissures in molars and bicuspids were made with a rat-tail file.

This establishment was subsequently moved to Race Street above Eighth. The writer of this retains, as a boy, a very vivid recollection of this humble start of the S. S. White Manufacturing Company, as well as that of the business place of the uncle that preceded it.

Thus for fifty years, from fifteen to sixty-five, the subject of this sketch was connected with the business, and was an active participator in the growth of the dental profession from its mechanical side from that period until the present.

Dr. James W. White married, October 28, 1847, Mary Ann, daughter of James and Maria McClaranan. He leaves, besides his widow, three sons,—Dr. J. William White, Samuel S. White, and Louis P. White, neither of whom follow the work of the father, the former being a prominent surgeon, the second a printer, of the firm of Patterson & White, printers of the *Dental Cosmos*, and the third a wholesale jeweller.

Dr. White was a member of the firm of Hance Brothers & White, Manufacturing Chemists.

The degree of A.M. was conferred on him by the St. Lawrence University, of Canton, N. Y., the degree of M.D. was received at the Department of Medicine of the University of Pennsylvania, and that of D.D.S. (honorary) at the Pennsylvania College of Dental Surgery.

The activity of mind, always a feature of his character, very early brought him into association with the best intellectual thought of Philadelphia. He was made secretary of the People's Literary Institute at the age of thirty-four, and was active in bringing to the city some of the ablest platform speakers, among them Beecher, Chapin, Curtis, Sumner, etc. This was at a period of great agitation on the slavery question, and a time when it required courage of a high order to maintain an unpopular position; but Dr. White never hesitated, and in a controversy with the then mayor of Philadelphia (Henry), he exhibited the same vigor in the defence of free speech as he at a later period demonstrated in his historical conflict with Mayor Fitler.

He was active in many public charities. The Maternity Hospital is due to his energy, and he remained at its head to the end. The Freedman's Aid Association claimed his interest, and when the great Sanitary Fair was organized, during the war, he was made chairman of the Committee on Orations and Lectures, and realized the handsome sum of ten thousand dollars for that memorable effort.

At the reorganization of the municipal government, he was made president of the Department of Charities of Philadelphia under Mayor Fitler. This appointment was received with expressions of universal satisfaction; but he remained only a portion of the term, owing to disagreements between himself and his superior in office, and he left this important position with the regret of his fellow-citizens. Denominationally, he was a member of the Universalist Church, in which he was moderator for many years, and also was superintendent of the Sunday-school.

Of later years, he was present at all the large gatherings of the dental profession, and, although not a member of any of these organizations, he exerted a great influence socially, and his genial presence will be greatly missed at their annual convocations.

As president of probably the largest organization for the manufacture and sale of dental articles, and as editor of the *Dental Cosmos*, he will be best known to the profession. This periodical has, under his management, increased not only its reputation, but has become, as its name implies, a world's journal in its special line of thought.

All of those who laid the foundation of the house of the S. S. White Manufacturing Company have now passed away, but it has gone beyond individual limitations, and will continue as a monument to the energy and far-seeing abilities of Dr. S. S. White and his brother James.

He died in full harness, for he remained later than usual at his place of business on Tuesday the 26th, and early Wednesday morning he passed into the unseen. The immediate cause of death was heart-failure.

The funeral was attended by a large number of persons prominent in various walks of life. He was buried May 29, at Woodlands Cemetery, Philadelphia.

